# Radio Electric Asymmetric Conveyer Reparative Effects on Muscle Injuries: A Report of Two Cases

**DOI:** 10.7759/cureus.24904

**Published:** 2022-05-11

**Authors:** Alessandro Castagna, Vania Fontani, Salvatore Rinaldi

**Affiliations:** 1 Department of Regenerative Medicine, Rinaldi Fontani Institute, Florence, ITA; 2 Department of Research, Rinaldi Fontani Foundation, Florence, ITA

**Keywords:** reparative medicine treatments, regenerative medicine treatments, reac, biostimulation, tear, injury, muscle

## Abstract

Cells and tissues work like batteries, positively charged by potassium ions and negatively charged by chloride ions. The difference in potential gradient generates an ionic flux, and this, in turn, generates a current that develops endogenous bioelectric fields (EBFs), which are fundamental for all cellular life processes, including reparative phenomena. In damaged tissues, the ionic flow is altered and, consequently, the production of EBFs is altered. This determines an alteration of the reparative processes. In previous studies, the reparative and regenerative treatments of radio electric asymmetric conveyer (REAC) technology have been shown to favor and accelerate the reparative processes of injured tissues, inducing the recovery of ionic flows and EBFs. The purpose of this report is to illustrate the clinical efficacy of REAC treatments for reparative tissue optimization on muscle injuries, even in those with a severity of third degree.

## Introduction

Muscle tears are frequent injuries, especially in athletes. These injuries are classified into degrees [1].

In a first-degree tear, the laceration of a few myofibrils within a muscle bundle occurs but the entire bundle is not lacerated [2]. In a second-degree tear, the laceration of one or more muscle bundles occurs, only considering cases involving less than 3/4 of the anatomical sectional surface of the muscle in the area. Clinically, the functional deficit is important but not absolute [2]. In a third-degree tear, there is a break of the muscle continuity, which involves more than 3/4 of the surface of the anatomical section of the muscle in the area. Clinically, the functional deficit is practically absolute [2].

A third-degree tear can be further distinguished into a partial or total tear, depending on whether the laceration of the muscle section is massive but still incomplete or complete laceration of an entire muscle occurs.

In the two-three days following the injury, the most commonly used treatment is the R.I.C.E. approach [3], consisting of a sequence of rest, ice, compression, and elevation. Although not yet supported by scientific evidence, the R.I.C.E. approach is very popular [4]. In addition to this initial approach, anti-inflammatory medicines and painkillers are prescribed, if necessary. Physiotherapy is always recommended, which is often associated with laser treatments. Although widely used, these treatments seem to have no scientific evidence of efficacy [5,6].

As one of the technologies used to promote tissue repair, the radio electric asymmetric conveyer (REAC) technology has specific treatment protocols for this purpose. The most used is called tissue optimization-reparative (TO-RPR). The mechanism of action of the REAC technology is based on the recovery and optimization of the correct endogenous bioelectric charges within the injured tissues or endogenous bioelectric fields (EBFs) [7]. The REAC functional recovery of the correct EBF activity is able to promote reparative [7,8] and regenerative processes [9-11] and determine phenomena of direct cellular reprogramming [12-15].

## Case presentation

Case 1

**Figure 1:**
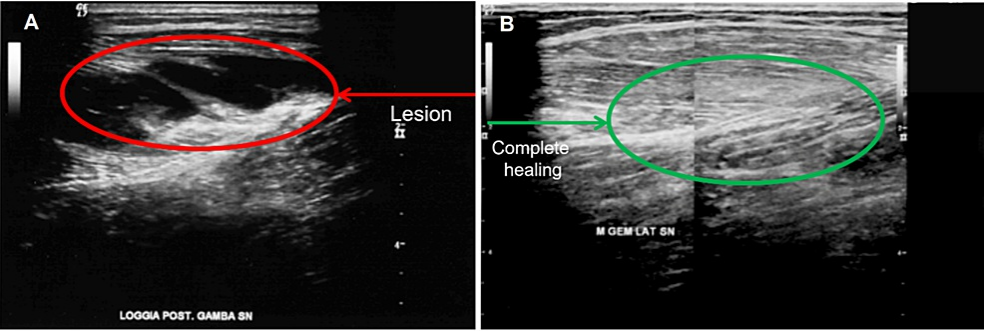
Case 1: third-degree tear of the lateral gastrocnemius muscle Third-degree tear of the lateral gastrocnemius muscle before (A) and after (B) 18 sessions of tissue optimization-reparative (TO-RPR) with radio electric asymmetric conveyer (REAC) technology. Each session lasted 30 minutes, three sessions per day for six days.

The first patient was a 47-year-old man. During a tennis match on November 22, the patient reported a muscle lesion. On the same day, the patient underwent an ultrasound scan which allowed for the diagnosis of a third-degree muscle lesion of the lower middle third of the lateral gastrocnemius (Figure 1A). The patient came for our observation one week after the trauma and started the REAC TO-RPR treatment 10 days after the trauma.

**Figure 2:**
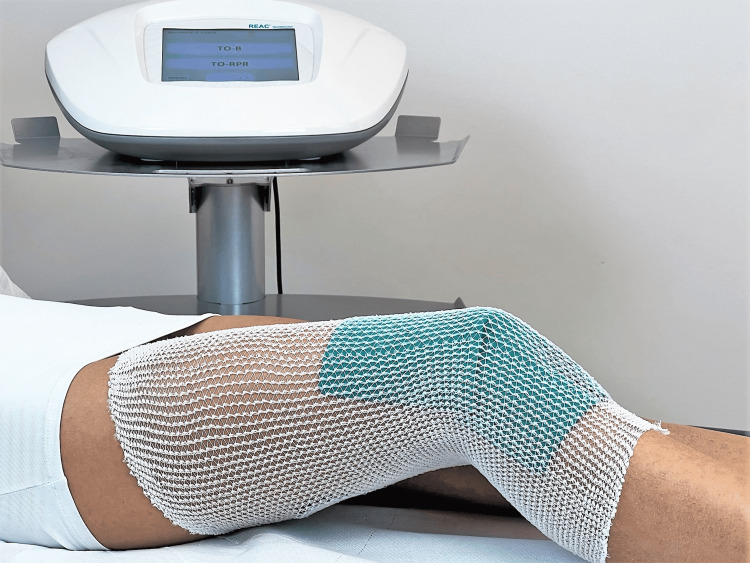
Example of administration of the TO-RPR treatment TO-RPR: tissue optimization-reparative

Each REAC TO-RPR treatment session lasted 30 minutes, according to the standardized procedure. The treatment parameters are preprogrammed and cannot be changed by the operator. The treatment is administered by placing an asymmetric conveying probe (ACP) on the area to be treated. The ACP is kept on the area by means of a tubular elastic gauze (Figure 2) and is connected to the REAC device, BENE Model 110 (therapeutic electromedical equipment for neurobiological stimulation CE 1282; ASMED SRL, Florence, Italy). The REAC TO-RPR cycle was administered in three sessions per day for six days. In the following February, the patient underwent a control ultrasound, which showed complete healing of the lesion (Figure 1B).

Case 2

The second patient was a 47-year-old, male, semi-professional soccer player. In June, he suffered a trauma on his medial gastrocnemius during a soccer match.

**Figure 3:**
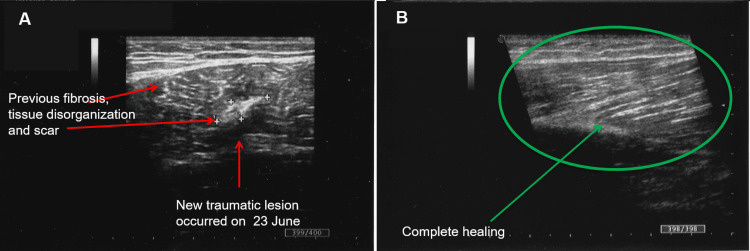
Before (A) and after (B) 18 REAC TO-RPR sessions (15 minutes for each session), three sessions per day for six days REAC: radio electric asymmetric conveyer; TO-RPR: tissue optimization-reparative

On the same day, he had an ultrasound, which revealed a second-degree lesion in a previously damaged tissue with fibrosis, tissue disorganization, scarring, and the presence of multiple microcalcifications (Figure 3A). The patient came for our observation one day after the trauma and started the REAC TO-RPR treatment on the same day.

In this case, each REAC TO-RPR treatment session lasted 15 minutes using the same device and the same method of administration as in the previous case. The REAC TO-RPR cycle was administered in three sessions per day for six days. In the following July, the patient underwent a control ultrasound, which showed complete healing of the lesion (Figure 3B).

## Discussion

Although the use of artificially produced electricity is relatively recent, the effects of electricity produced by some organisms, especially fish, had already been appreciated in the past to promote wound healing [16]. The regenerative potential of injured tissues is closely linked to the intensity of injury-induced endogenous bioelectric fields (EBFs), which are necessary and sufficient to stimulate regeneration [17-19]. This knowledge has led biotechnological research to try to restore the activity of ionic flows, altered by the concentration of charges that are formed inside the damaged tissues [17]. The restoration of the functional electrotaxis allows the repair of damaged tissues [20]. In fact, the alteration of the concentration of charges prevents the creation of functional ionic flows and, therefore, the creation of the EBFs essential to favor the reparative processes in any type of tissue [17,19].

The results presented in this case report highlight how the REAC TO-RPR treatment can accelerate the repair process and facilitate the healing of damaged tissue with better quality tissue repair. This positive effect is also evident in the reduction/disappearance of the calcified formations present in the second case.

## Conclusions

In the two cases described in this manuscript, we showed how the neurobiological stimulation of REAC TO-RPR treatment can restore the ion fluxes and consequently optimize the EBFs, which are crucial in the reparative processes. This positive effect is evident in the almost total restoration of the normal characteristics of the muscle tissue. These observations, previously detected in other tissues, allow us to envisage other uses of this therapeutic tool to improve the reparative processes and the quality of life of subjects affected by muscle injuries
